# Aplysin Sensitizes Cancer Cells to TRAIL by Suppressing P38 MAPK/Survivin Pathway

**DOI:** 10.3390/md12095072

**Published:** 2014-09-25

**Authors:** Jia Liu, Leina Ma, Ning Wu, Ge Liu, Lanhong Zheng, Xiukun Lin

**Affiliations:** 1 Institutes of Oceanology, Chinese Academy of Sciences, Qingdao 266071, China; E-Mails: dadaliujia@gmail.com (J.L.); wuning@qdio.ac.cn (N.W.); liug878@163.com (G.L.); 2 Graduate School, University of Chinese Academy of Sciences, Beijing 100049, China; 3 Department of Molecular Biology, School of Medicine and Pharmacy, Ocean University of China, Qingdao 266003, China; E-Mail: leinama@gmail.com; 4 Yellow Sea Fisheries Research Institute, Chinese Academy of Fishery Sciences, Qingdao 266071, China; 5 Department of Pharmacology, Capital Medicine University, Beijing 100069, China

**Keywords:** aplysin, TRAIL, p38 MAPK, survivin

## Abstract

TNF-related apoptosis-inducing ligand (TRAIL) is a tumor-selective apoptosis inducer and has been shown to be promising for treating various types of cancers. However, the application of TRAIL is greatly impeded by the resistance of cancer cells to its action. Studies show that overexpression of some critical pro-survival proteins, such as survivin, is responsible for TRAIL resistance. In this study, we found that Aplysin, a brominated compound from marine organisms, was able to restore the sensitivity of cancer cells to TRAIL both *in vitro* and *in vivo*. Aplysin was found to enhance the tumor-suppressing capacity of TRAIL on several TRAIL-resistant cancer cell lines. TRAIL-induced apoptosis was also potentiated in A549 and MCF7 cells treated with Aplysin. Survivin downregulation was identified as a mechanism by which Aplysin-mediated TRAIL sensitization of cancer cells. Furthermore, the activation of p38 MAPK was revealed in Aplysin-treated cancer cells, and its inhibitor SB203580 was able to abrogate the promoting effect of Aplysin on the response of cancer cells to TRAIL action, as evidenced by restored survivin expression, elevated cell survival and reduced apoptotic rates. In conclusion, we provided evidence that Aplysin acts as a sensitizer for TRAIL and its effect on p38 MAPK/survivin pathway may partially account for this activity. Considering its low cytotoxicity to normal cells, Aplysin may be a promising agent for cancer treatment in combination with TRAIL.

## 1. Introduction

TNF-related apoptosis-inducing ligand (TRAIL) is a cytokine that specifically elicits programed cell death, apoptosis, in tumor cells. The fact that it causes no significant cytotoxicity to normal cells makes it a promising agent for clinical application. Mechanistically, TRAIL binds and activates death receptors, TRAILR1 or TRAILR2, located in cytoplasm membrane of cancer cells. In turn, activated receptors are cross-linked and induce dimerization of procaspase-8. Caspase cascades were activated, and as a consequence, cells undergo apoptosis [1]. Because of its tumor-specific apoptosis-inducing property, TRAIL has served as a biological agent of cancer treatment for decades [2], however, clinical trials have not shown significant survival benefit on cancer patients [3].

The primary cause of poor outcomes of TRAIL-mediated cancer therapy in patients is probably due to resistance of cancer cells to TRAIL [4]. Using various cancer cell lines as models, researchers have gotten insight into the mechanisms by which cancer cells become not sensitive to TRAIL. To escape the action of TRAIL produced from tumor microenvironments, cancer cells develop resistance to TRAIL through multiple mechanisms, including reducing the expression of TRAIL receptors, increasing the expression of TRAIL decoy receptors or disrupting the effectors downstream of TRAIL signaling [4]. To change the response of intracellular molecules to TRAIL action, cancer cells minimize the expression of caspase proteins or elevate the level of pro-survival proteins, such as xIAP, cIAP or survivin [5]. Decreasing the level of intracellular survivin was sufficient to sensitize cancer cells to TRAIL [6,7,8]. Multiply natural or synthetic compounds have been demonstrated to increase the responsiveness of cancer cells to TRAIL action by depleting survivin [9,10,11,12,13].

**Figure 1:**
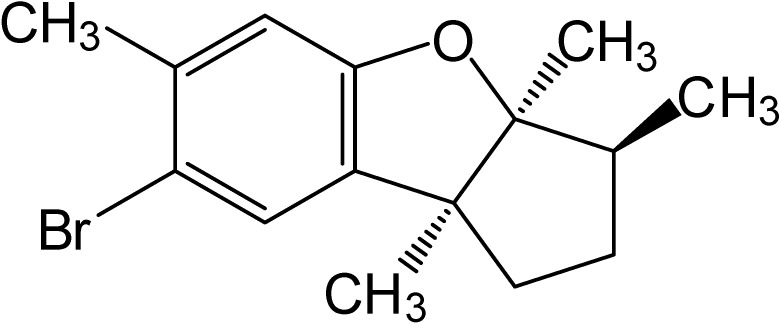
The structure of Aplysin.

Aplysin (C_15_H_19_OBr) is a seaweed bromo sesquiterpene compound from laurencia tristicha with a molecular weight of 295 (Figure 1). Previous study has shown that aplysin can reduce ethanol-induced hepatic injury in mice [14]. However, its potential application for cancer therapy has not been explored yet.

In this study, we found that Aplysin was able to enhance the anti-tumor activity of TRAIL on resistant cancer cell lines. And p38 MAPK-dependent survivin downregulation in cancer cells is required for the synergistic effect of Aplysin on TRAIL action.

## 2. Results and Discussion

### 2.1. Aplysin Increases Antitumor Activity of TRAIL on Cancer Cells in Vitro

**Figure 2:**
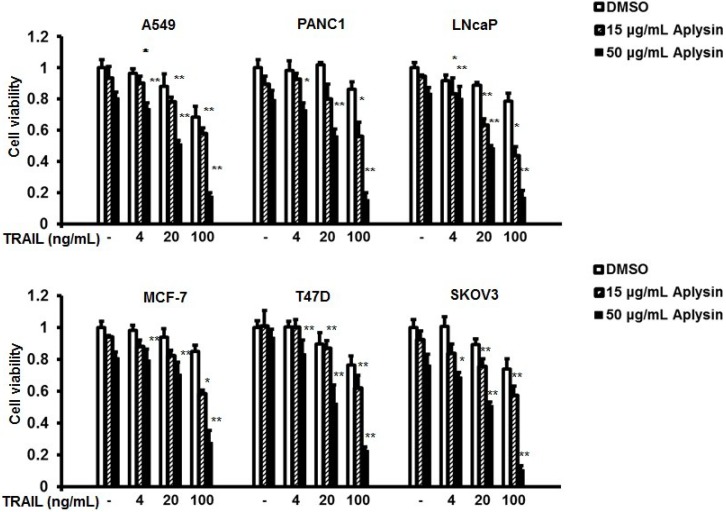
Aplysin enhanced the sensitivity of cancer cells to TRAIL. MCF-7, T47D, SKOV3, A549, PANC-1, and LNcaP cells were treated with Aplysin (15 or 50 μg/mL) or/and TRAIL (4, 20 or 100 ng/mL). 24 h later, MTT assays were performed to determine their viabilities. (* *P* < 0.05; ** *P* < 0.01).

The structure of Aplysin is shown in Figure 1. TRAIL-resistant cell lines, MCF-7, T47D, SKOV3, A549, PANC1, and LNcaP, were used to study the effect of Aplysin on their sensitivity to TRAIL. Treatment of cancer cells with TRAIL alone slightly suppressed the viability of these cells with an inhibition rate of 30% at a concentration of 100 ng/mL (Figure 2). However, Aplysin treatment was able to significantly enhance their sensitivity to TRAIL. Especially, combined treatment of 50 μg/mL Aplysin and 100 ng/mL TRAIL suppressed cell survival by 70%–90% for most of the tested cells (Figure 2). Also, the promoting effect of Aplysin on TRAIL action depended on the concentration of Aplysin. In addition, immunoblot analysis of survivin, XIAP, cFLIP, FADD, TRAIL-R1 (DR4) and TRAIL-R2 (DR5) was performed in these cancer cells (Supplementary Figure S1).

### 2.2. Aplysin Enhances the Inhibitory Effect of TRAIL on the Growth of Tumor Xenografts in Mice

**Figure 3:**
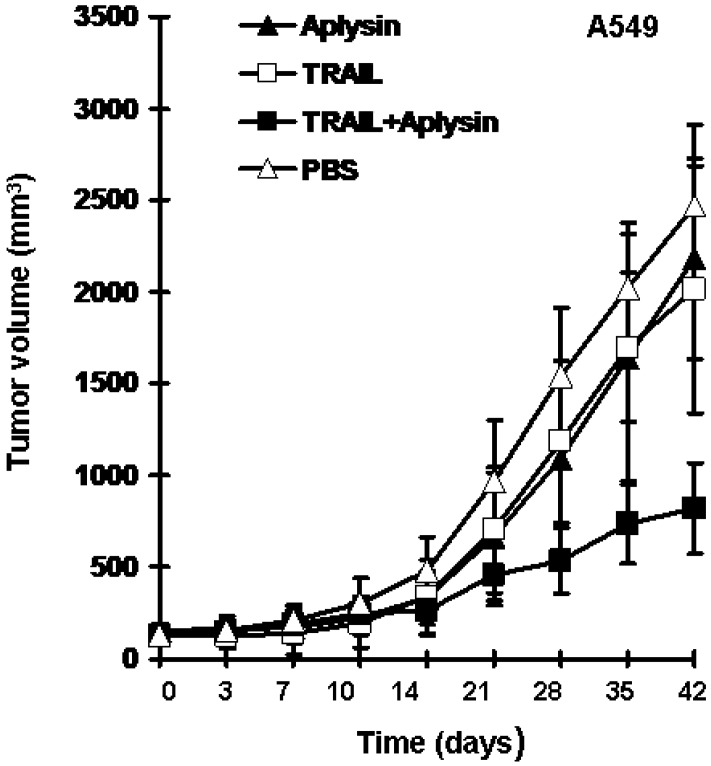
Aplysin enhanced the inhibitory effect of TRAIL on the growth of tumor xenografts in mice. A549 cells were used to establish a tumor xenograft mouse model. TRAIL (200 μg/kg) or/and Aplysin (150 mg/kg) were administrated intratumorally and by gavage, respectively. The tumor volumes were shown as mean ± SD (*n* = 6).

To further confirm *in vivo* enhancing effect of Aplysin on TRAIL action on cancer cells, we established a mouse model bearing A549 tumor xenografts. The results revealed that Aplysin or TRAIL alone slightly suppressed the growth of tumors; TRAIL at a concentration of 200 μg/kg or Aplysin at 150 mg/kg resulted in an inhibition rate of 11.76% and 18.45% respectively. Whereas the combination of Aplysin and TRAIL exhibited synergistic inhibitory effect on tumor growth with an inhibition rate of 66.75% (Figure 3). The results of animal experiments suggested that there exists synergistic effect of the combination of TRAIL and aplysin; Aplysin can increase the sensitivity of cancer cells to TRAIL action *in vivo*.

### 2.3. Aplysin Enhances Apoptosis-Inducing Activity of TRAIL on A549 and PANC1 Cancer Cells

**Figure 4:**
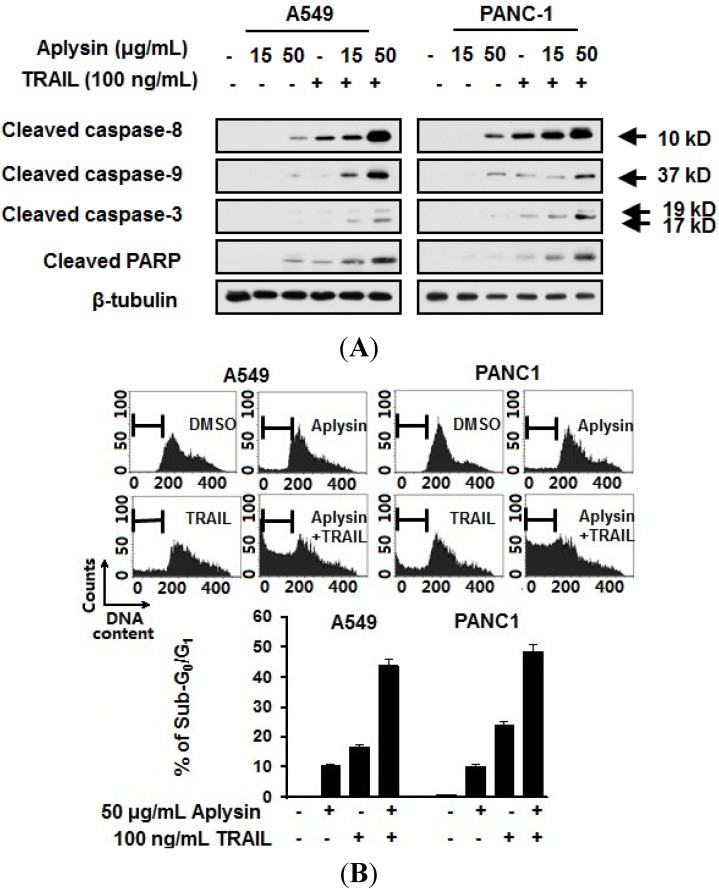
Aplysin potentiated the apoptosis in cancer cells treated with TRAIL. A549 and PANC-1 cells were treated with Aplysin (15 or 50 μg/mL) or/and TRAIL (100 ng/mL). After cultured for 24 h, the expression of cleaved caspase-3, caspase-8, caspase-9 and PARP were detected by immunoblot analysis (**A**); and cytometrical analysis of sub-G_0_/G_1_ population (**B**).

It has been well demonstrated that TRAIL induces apoptotic events in cancer cells. Therefore, we detected the effect of Aplysin on the activation of apoptotic pathway in cancer cells treated with Aplysin. Aplysin was revealed to increase the cleavage of caspase-8, -9 and -3 thereby enhancing the activity of apoptosis-promoting protease in A549 and PANC1 cells (Figure 4A and Figure S6A). Also, more cytochrome C was released into cytosol fraction under the co-treatment of Aplysin and TRAL (Figure 4A). Poly ADP ribose polymerase (PARP), the biomarker of cellular apoptosis, was highly cleaved under this stimulation (Figure 4A).

Furthermore, the apoptotic rates were significantly increased in cells treated with the combination of TRAIL and Aplysin, as determined by flow cytometry. The percentages of sub-G_0_/G_1_ population were 43.74% and 48.24%, respectively, in A549 and PANC1 cells exposed to 50 μg/mL Aplysin and 100 ng/mL TRAIL respectively, compared with Aplysin alone (A549, 10.52%; PANC1, 10.18%) or TRAIL alone (A549, 10.52%; PANC1, 23.99%) (Figure 4B). Pan-caspase inhibitor Z-VAD-FMK was able to prevent the synergistic cytotoxicity of Aplysin and TRAIL (Supplementary Figure S2). These data suggested that Aplysin promotes the apoptosis-inducing activity of TRAIL on cancer cells.

The inherited and acquired resistance of cancer cells to TRAIL action severely hampered its application. Therefore, much effort has been made to sensitize cancer cells to TRAIL-induced apoptosis. Fuligocandin B, a cycloanthranilylproline derivative, enhanced the sensitivity of leukemic cells to TRAIL action [15]. Eupatolide was also found to sensitize breast cancer cells to TRAIL-stimulated apoptosis [16]. Two compounds from *Pongamia pinnata* was able to overcome TRAIL resistance of gastric carcinoma cells [17]. For this first time, we provided evidences that Aplysin is also a potent sensitizer for TRAIL to induce apoptosis in cancer cells, in this study.

### 2.4. Survivin Is Downregulated by Aplysin Treatment in TRAIL-Resistant Cancer Cells

Many research groups have been focusing on the molecular mechanisms by which cancer cells become insensitive to TRAIL-induced apoptosis. TRAIL exerts its proapoptotic activity by activating caspase pathways [18]. As endogenous inhibitors of caspase pathway, the abnormality of inhibitors of apoptosis (IAP) family, which includes XIAP, c-IAP1, c-IAP2, NIAP and survivin, is thought to affect the sensitivity of cells to TRAIL action [4]. Many studies have shown that elevated level of survivin in cancer cells has been demonstrated to be one of major mechanisms responsible for the insensitivity of cancer cells to TRAIL action [4]. Thus, decreasing survival expression appeared to be an effective strategy to overcome the resistance of cancer cells to TRAIL.

**Figure 5:**
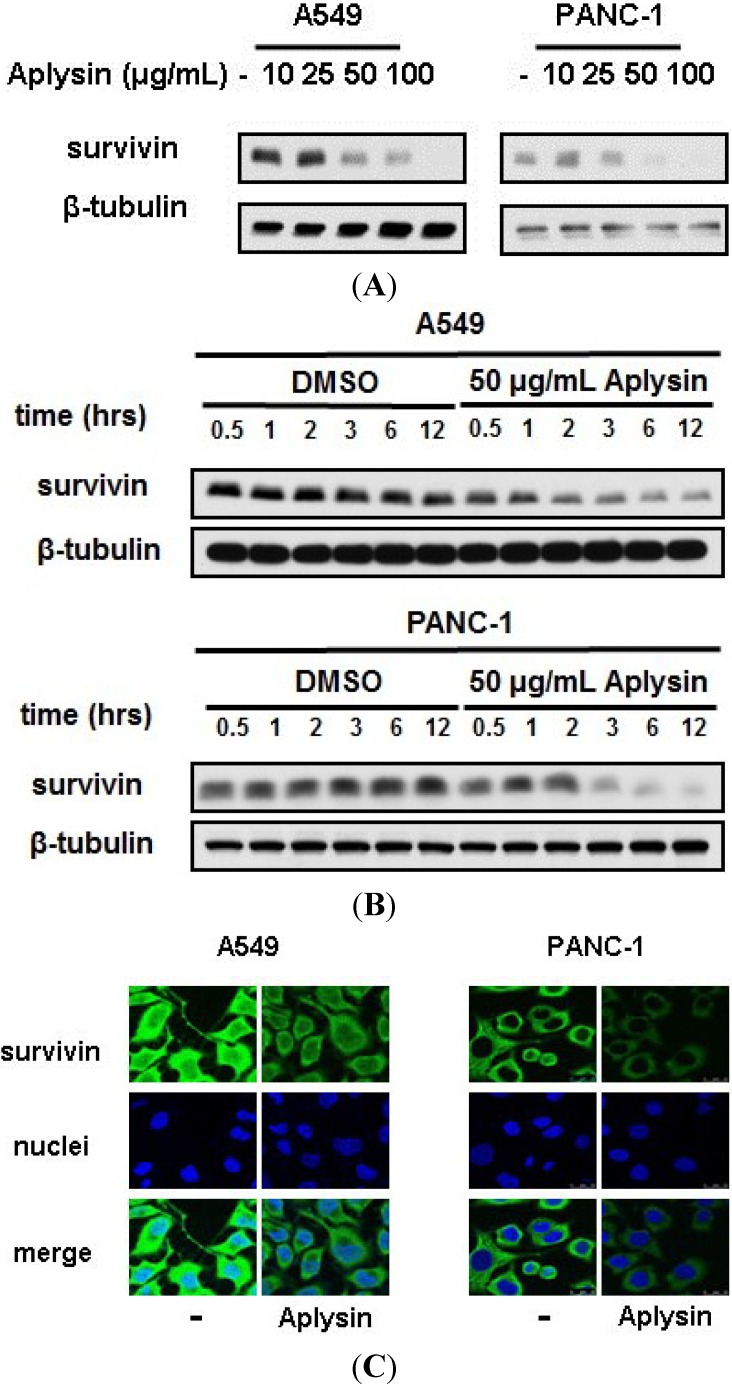
Aplysin suppressed the expression level of survivin in cancer cells in a dose- and time-dependent manner. (**A**) Aplysin (10, 25, 50 and 100 μg/mL) was added to the cultures of A549 and PANC-1 cells. After cultured for 12 h, survivin levels were evaluated with immunoblot assays; (**B**) Aplysin (50 μg/mL) was added to the cultures of A549 and PANC-1 cells. Survivin expression was determined using immunoblot assays at the indicated time points. DMSO was used as negative control; (**C**) A549 and PANC-1 cells were treated with 50 μg/mL Aplysin for 12 h, followed by immunofluorescent staining to confirm survivin levels in cells.

Considering that reduction in survivin expression is one of mechanisms by which cancer cells resist against TRAIL action, we detected the expression level of survivin in Aplysin-treated cancer cells. Immunoblot analysis revealed that the survivin level was greatly reduced in cancer cells with Aplysin stimulation, both in dose- and time-dependent manner (Figure 5A,B and Supplementary Figure S3). This change was also confirmed by immunofluerescent staining (Figure 5C), shown as diminished signaling intensity of survivin-targeting antibody in A549 and MCF7 cells with Aplysin stimulation.

### 2.5. The Decrease in Survivin Expression Level Is Responsible for Aplysin-Caused Cancer Cell Sensitization to TRAIL

**Figure 6:**
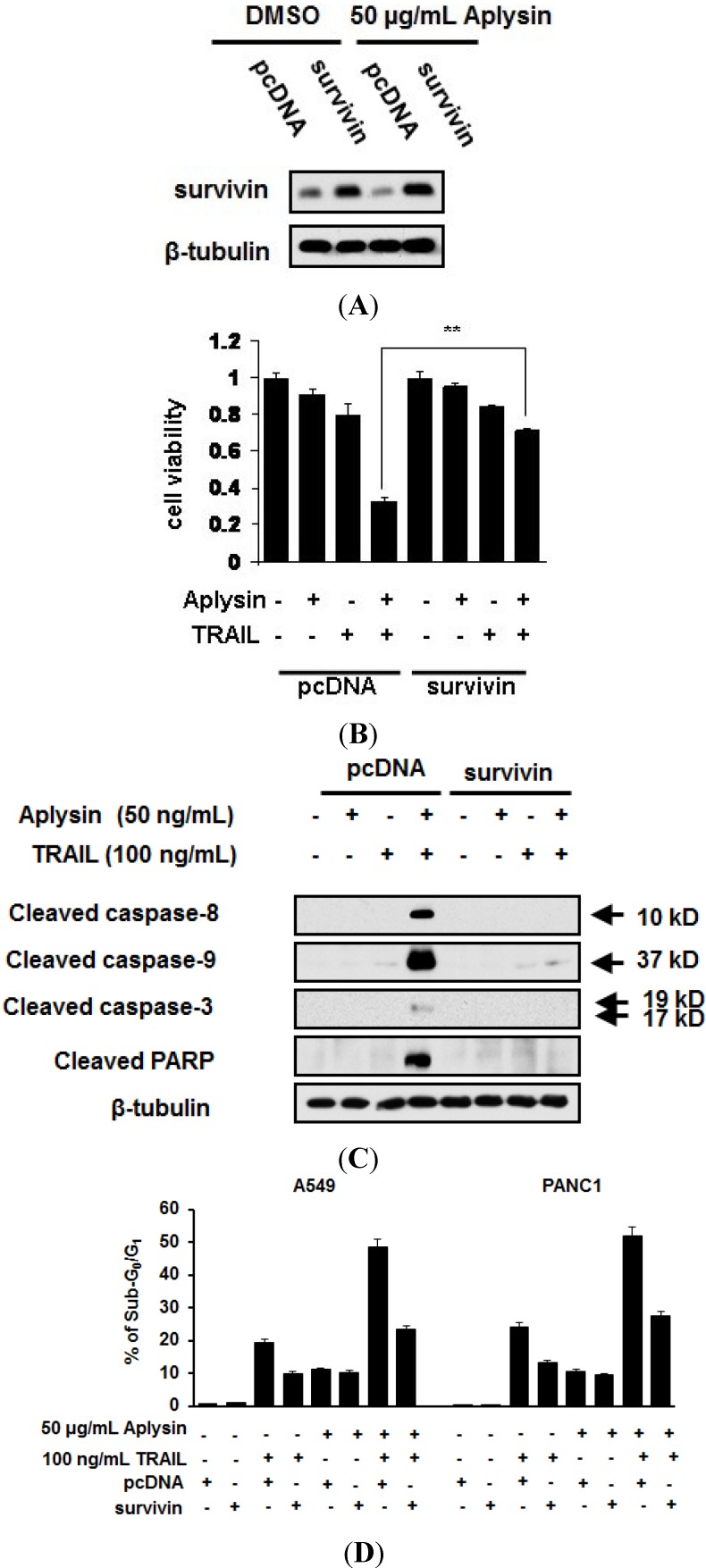
Survivin downregulation was required for the promoting effect of Aplysin on the sensitivity of cancer cells to TRAIL. (**A**) A549 cells were transfected with pcDNA-survivin (survivin) using Lipofectamine 2000. After cultured for 24 h, the cells were treated with 50 μg/mL Aplysin for another 12 h. Then, survivin expression levels were detected by immunoblot assays. pcDNA3.1 (pcDNA) was used as control; (**B**) A549 and PANC-1 cells were transfected with pcDNA-survivin (survivin). After cultured for 24 h, the cells were treated with 50 μg/mL Aplysin for another 24 h. Then, MTT assays were performed to determine their viability; (**C**) A549 cells were transfected with pcDNA-survivin (survivin). After incubation for 24 h, the cells were treated with 50 μg/mL Aplysin for 12 h, followed by immunoblot analysis of caspase expression; (**D**) A549 cells were transfected with pcDNA-survivin (survivin). After cultured for 24 h, the cells were treated with 50 μg/mL Aplysin or/and 100 ng/mL TRAIL for 12 h, followed by cytometrical analysis of sub-G_0_/G_1_ population.

To determine the effect of reduced survivin expression in Aplysin-induced sensitization of cancer cells to TRAIL, we restored survivin expression via transfecting A549 and PANC1 cells with pcDNA-survivin (Figure 6A). pcDNA-survivin transfection partially abrogated Aplysin effect on the action of TRAIL on cancer cell, as evidenced by increased survival of A549 and PANC1 cells, and their reduced sub-G_0_/G_1_ population when treated with both Aplysin and pcDNA-survivin (Figure 6B,D). The role of reduced expression of survivin in Aplysin-mediated TRAIL sensitization was further confirmed by immunoblot analysis of apoptosis-related proteins, such as caspase-8, -9 and -3 (Figure 6C and Supplementary Figure S6B).

A number of anticancer agents are reported to enhance the sensitivity of cancer cells to TRAIL via downregulation of survivin. SHetA2, a flexible heteroarotinoid, was proven to enhance the sensitivity of lung cancer cells to TRAIL-elicited apoptosis through suppressing survivin expression [19]. Bufotalin-mediated sensitization of HeLa cells to TRAIL action was also found to be associated with the downregulation of survivin expression [20]. Recently, another natural compound, AW00178, was also reported to reduce survivin level to increase the sensitivity of lung carcinoma cells to TRAIL-mediated apoptosis [21]. In this study, we showed that Aplysin was also able to suppress the expression of survivin, according to the data from immunoblotting and immunofluorescent staining assays. The reduced in survivin expression level mediated the effect of Aplysin on the sensitivity of cells to TRAIL. However, Aplysin may not enhance TRAIL action in resistant cancer cells with low basal level of survivin, because these cells were believed to prevent the proapoptotic effect of TRAIL by changing the abundance of TRAIL receptors.

### 2.6. Aplysin-Induced Survivin Downregulation Was Partially Mediated by p38 MAPK Signaling

**Figure 7:**
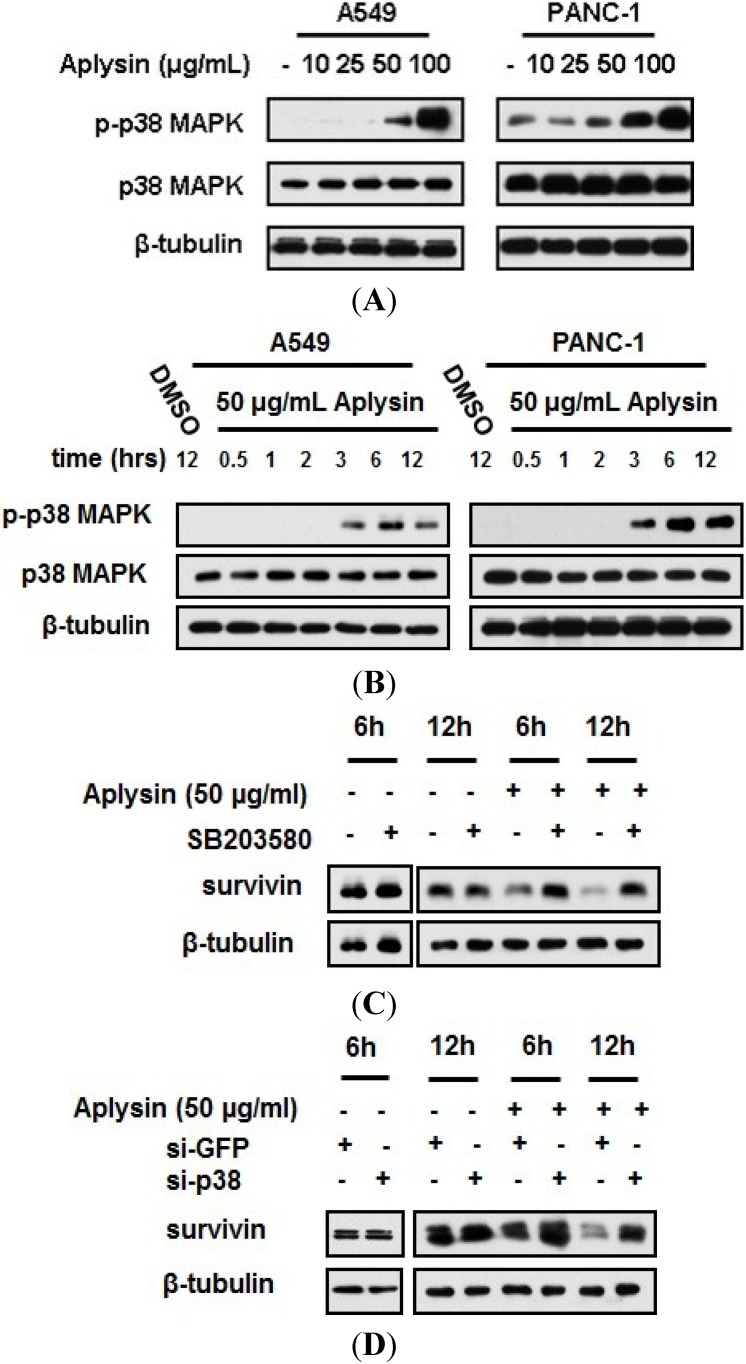
p38 MAPK activation was essential for Aplysin suppression of survivin expression. (**A**) Aplysin (10, 25, 50 and 100 μg/mL) was added to the cultures of A549 and PANC-1 cells. After cultured for 12 h, p38 MAPK phosphorylation were evaluated with immunoblot assays; (**B**) Aplysin (50 μg/mL) was added to the cultures of A549 and PANC-1 cells. The expression of phosphorylated p38 MAPK was determined using immunoblot assays at the indicated timepoints. DMSO was used as negative control; (**C**) A549 cells were pretreated with SB203580 (20 μM) for 1 h prior to Aplysin (50 μg/mL) incubation. After cultured for 6 h or 12 h, the cells were subjected to the immunoblot analysis of survivin expression; (**D**) A549 cells was transfected with small interfering RNA, si-GFP or si-p38, and 24 h later, Aplysin (50 μg/mL) was added to the media for 6 h or 12 h stimulation. Survivin expression was detected by immunoblot assays.

It has been documented that p38 MAPK activation, by Trichostatin A, was able to suppress survivin mRNA transcription in colon cancer cells [22]. Thus, we used immunoblot assay to detect the change in p38 MAPK activation in cancer cells under Aplysin treatment. The phosphorylation level of p38 MAPK was increased in cancer cells treated with Aplysin, in a dose- and time-dependent manner (Figure 7A,B and Supplementary Figure S4). p38 MAPK inhibitor, SB203580, and p38 MAPK-specific siRNA partially prevented cancer cells from Aplysin-induced survivin downregulation (Figure 7C,D). These data showed that p38 MAPK activation partially mediated the reduction in survivin expression induced by Aplysin.

However, we found that survivin level began to decline after treatment of Aplysin for 3 h (Figure 5B), while Aplysin failed to trigger the phosphorylation of p38 MAPK until 3 h treatment of Aplysin (Figure 7B). Thus, there is a possibility that Aplysin perhaps affected the expression of survivin via a p38MAPK-independent way. We also detected the expression levels of apoptosis inhibitors, XIAP and cFLIP, TRAIL receptors, TRAIL-R1 and TRAIL-R2, as well as TRAIL decoy receoptor, TRAIL-R3 and TRAIL-R4, in cancer cells treated with Aplysin. But no significant difference was observed (Supplementary Figure S5).

### 2.7. Aplysin-Induced p38 MAPK Activation Was Partially Required for the Sensitization of Cancer Cells to TRAIL

**Figure 8:**
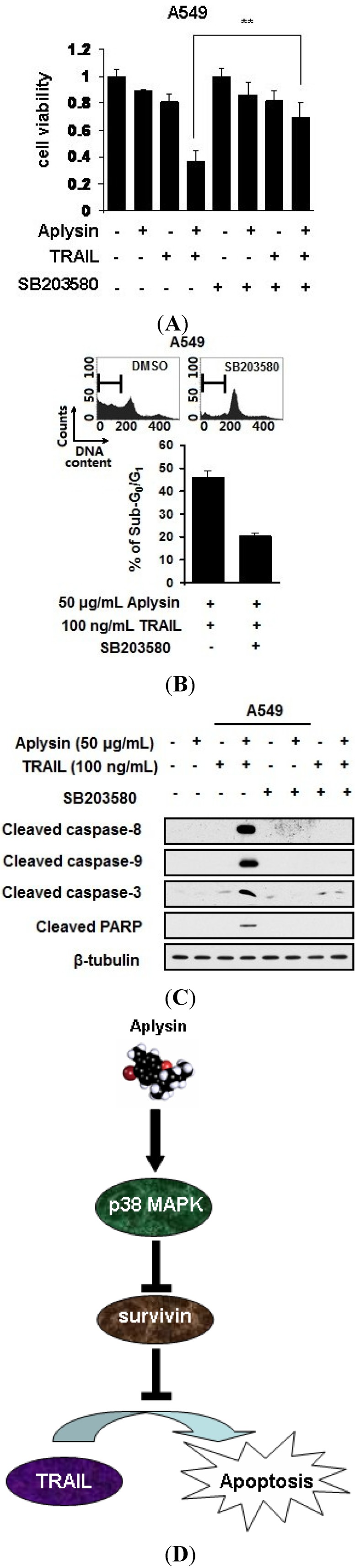
The effect of Aplysin on TRAIL sensitivity of cancer cells depends on p38 MAPK activation. (**A**) A549 cells were pretreated with 20 μM SB203580 for 1 h prior to the co-treatment of Aplysin (50 μg/mL) and TRAIL (100 ng/mL). After culutured for 24 h, the cell survival was assessed by MTT assays. A549 cells were subjected to the above treatments. The cells also underwent cytometrical analysis to determine the sub-G_0_/G_1_ population after treating the cells for 12 h (**B**) and immunoblot analysis of apoptosis-related markers, cleaved caspase-3, caspase-8, caspase-9 and PARP (**C**); (**D**) The molecular pathway associated with the promoting effect of Aplysin on TRAIL action was briefly shown. Aplysin triggered the activation of p38 MAPK in cancer cells, which in turn suppressed the transcription of survivin, an anti-apoptosis protein. Consequently, the proapoptotic activity of Aplysin was enhanced by this stimulation.

Subsequently, we used SB203580 to study the role of p38 MAPK activation in Aplysin-mediated sensitization of cancer cells to TRAIL action. p38 MAPK inhibition by SB203580 was able to increase the survival of cancer cells treated with Aplysin and TRAIL (Figure 8A). Furthermore, blocking p38 MAPK activation partially abrogated the stimulatory effect of Aplysin on the apoptotic response of cancer cells to TRAIL. SB203580 lead to a decrease in the percentage of sub-G_0_/G_1_ population in cancer cells treated with Aplysin and TRAIL (Figure 8B). Also, the cleavage of caspase family proteases was inhibited in SB203580-treated cancer cells under Aplysin and TRAIL co-stimulation (Figure 8C). The above results demonstrated that p38 MAPK activation is, at least in part, responsible for Aplysin-mediated sensitization of cancer cells to TRAIL action.

## 3. Experimental Section

### 3.1. Cell Culture

Human breast cancer cells MCF-7 and T47D, human ovarian cancinoma cell line SKOV3, human non-small cell lung cancer cell A549, human pancreatic carcinoma cell PANC1, and human prostate adenocarcinoma cell LNcaP were all purchased from American Type Culture Collection (ATCC, Manassas, VA, USA). These cells were cultured using the recommended media with 10% fetal bovine serume (FBS).

### 3.2. Chemical Reagents

Aplysin solutions were prepared following the previous procedures [23]. Briefly, three laurencia tristicha were provided and validated by the Institute of Oceanology, Chinese Academy of Sciences. The dried sample (5 kg) was soaked in 95% ethanol for 3 days and extracted 3 times to obtain 325 g extraction. The extract was fractioned with ethyl acetate to obtain 105 g extract. Then the ethyl acetate extract was further separated using silica gel column chromatography with petroleum ether-acetone as elution agent. The eluted fraction was then purified with repeat silica, bio-beads, Sephadex LH-20 column chromatography and reversed-phase HPLC. The obtained white compound was verified as aplysin (C_15_H_19_OBr) using NMR and mass spectrometry with a molecular weight of 295.

Aplysin was dissolved in DMSO to make a stock solution (10 mg/mL). The stock solution was diluted into the indicated concentration when used. Recombinant human soluble TRAIL was purchased from Peprotech (Rocky Hill, NJ, USA, Cat No. 310-04). Pan-caspase inhibitor Z-VAD-FMK was obtained for R & D (Minneapolis, MN, USA). Inhibitors that selectively blocked p38 MAPK, SB203580, was purchased from Cell Signaling Technology (Beverly, MA, USA, #5633). Cells were pretreated with SB203580 (10 μM) for 1 h prior to the stimulation with Aplysin.

### 3.3. Cell Viability Assay

Human cancer cells (5 × 10^3^) including MCF-7, T47D, SKOV3, A549, PANC-1, and LNcaP cells were seeded in each well of a 96-well plate. After incubation for 8 h, the cells were treated with DMSO, or certain concentration of Aplysin (15 μg/mL or 50 μg/mL) or/and recombinant human TRAIL protein (4 ng/mL, 20 ng/mL or 100 ng/mL). After cultured for another 24 h, cells were treated with 50 μL 3-(4,5-dimethylthiazol-2-yl)-2,5-diphenyltetrazolium bromide (MTT) (1 mg/mL) for 4 h incubation. MTT was removed and 150 μL DMSO was added. The spectrophotometric absorbance was measured on a model 550 microplate reader (Bio-Rad Laboratories, Hercules, CA, USA) at 570 nm with a reference wavelength of 655 nm. Cell viability was calculated according to the following formula: Cell viability rate = absorbance value of specific cells/absorbance value of control cells. Control cells were defined as the cells only treated with DMSO.

### 3.4. Animal Experiments

Human lung cancer A549 cells (5 × 10^6^) were subcutaneously injected to the flanks of 4–6 week old male nude BALB/c mice. The mice were randomly divided into 4 groups and each group contains 6 mice. Control group intratumorally administrated with PBS, TRAIL group treated with TRAIL (200 μg/kg), Aplysin group treated with Aplysin (150 mg/kg) and combination treated group intratumorally administrated with both TRAIL (200 μg/kg) and Aplysin (150 mg/kg). All of the mice were raised for 30 days. The tumor diameters were periodically measured with calipers. The volumes were calculated according to the following formula: Volume (mm^3^) = length × (width)^2^/2. No mice died of tumor loading in the experiments.

### 3.5. Apoptosis Assays

A549 and PANC-1 cells (2 × 10^5^) were seeded in each well of a 6-well plate. After incubation for 8 h, the cells were treated with DMSO, Aplysin (100 μg/mL) or/and TRAIL (100 ng/mL). After incubation for another 24 h, cells were harvested by centrifugation at 3000 *g* × 15 min, fixed with cold 70% ethanol and treated with RNase A (100 mg/L, Sigma, St. Louis, MO, USA) for 10 min. Finally, cells were stained with propidium iodide (PI, 50 mg/mL, Sigma, St. Louis, MO, USA) for cell cycle analysis by cell cytometry (FACSAria™ II, BD Biosciences, San Jose, CA, USA). Apoptotic rates were analyzed by counting sub-G_0_/G_1_ events in the euploid cell fraction.

### 3.6. Immunoblotting Assay

Proteins of cultured cells were harvested with M-PER Mammalian Protein Extraction Reagent (Thermo Scientific, Rockford, IL, USA), separated using 12% polyacrylamide gel electrophoresis and transferred onto 0.45 μm nitrocellulose membranes. The membranes were blocked with 5% fat-free dry milk in PBS and incubated with primary antibodies at 4 °C. Overnight, the membrane was incubated with corresponding secondary antibodies and visualized with SuperSignal West Dura Extended Duration Substrate (Thermo Scientific, Rockford, IL, USA). The antibodies involved in our study is as follows: Cleaved PARP, CST, #5625; Cleaved caspase-3 CST, #9664; Cleaved caspase-9, CST, #9501; Cytochrome C, GST, #11940; Cytochrome c oxidase subunit IV (COX IV) CST, #4850.

### 3.7. Vector Construction and Transfection

The procedure of vector construction was described previously [24]. Briefly, total cDNA obtained from SKOV3 cells was used as templates. The primers were as follows: forward: 5′-CCCAAGCTTATGGGTGCCCCGACGTTG-3′; reverse: 5′-GCTCTAGAACAGGCAGAAGCACCTCT-3′. The PCR product was purified using 10% agrose electrophoresis, and inserted into pcDNA-3.1 vector (Invitrogen, Carlsbad, CA, USA) at the sites of BamHI and XbaI to construct the plasmid pcDNA-survivin. pcDNA3.1 vector was used as the control.

A549 and PANC-1 cells (2 × 10^5^) were transfected with 3 μg pcDNA-survivin and pcDNA3.1 using Lipofectamin 2000 (Invitrogen, Carlsbad, CA, USA) according to the instructions of manufacturer. After incubation for 24 h, the cells were subjected to the subsequent experiments.

### 3.8. Statistical Analysis

The experiments except immunoblot assays were performed for at least three times. All values were reported as means ± SD, and compared at a given time point by unpaired, two-tailed t test. Data were considered to be statistically significant when *p* < 0.05 (*) and *p* < 0.01 (**).

## 4. Conclusions

Taken together, we demonstrated that Aplysin, a natural compound originating from oceans, can be used for cancer therapy by enhancing TRAIL action both *in vitro* and *in vivo*. p38 MAPK activation-dependent survivin underexpression is, at least in part, responsible for Aplysin’s activity. This study provides more evidence to show that sensitization of cancer cells to TRAIL may be served as a strategy in cancer therapy, and Aplysin possesses potential to be developed as a novel class of anticancer agents.

## Supplementary Files

Supplementary File 1
