# Supplementary material for: Aplysin Sensitizes Cancer Cells to TRAIL by Suppressing P38 MAPK/Survivin Pathway
Source: Mar Drugs. 2014 Sep 25;12(9):5072–88. doi: 10.3390/md12095072 (PMC4178493; doi:10.3390/md12095072)
Supplement: Supplementary File 1 [file marinedrugs-12-05072-s001.pdf]

Supplementary Information

Figure S1. The expression of survivin, XIAP, cFLIP, FADD, TRAIL-R1 (DR4) and TRAIL-R2 (DR5) was detected in the indicated cancer cells by immunoblot assays.

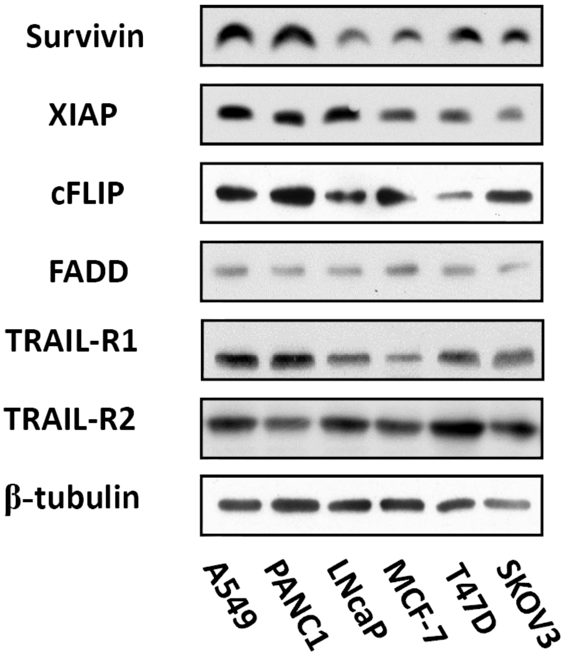

Figure S2. The viability of A549 and PANC1 cells were detected 24 h after Z-VAD-FMK (50 μM) was used by MTT assays.

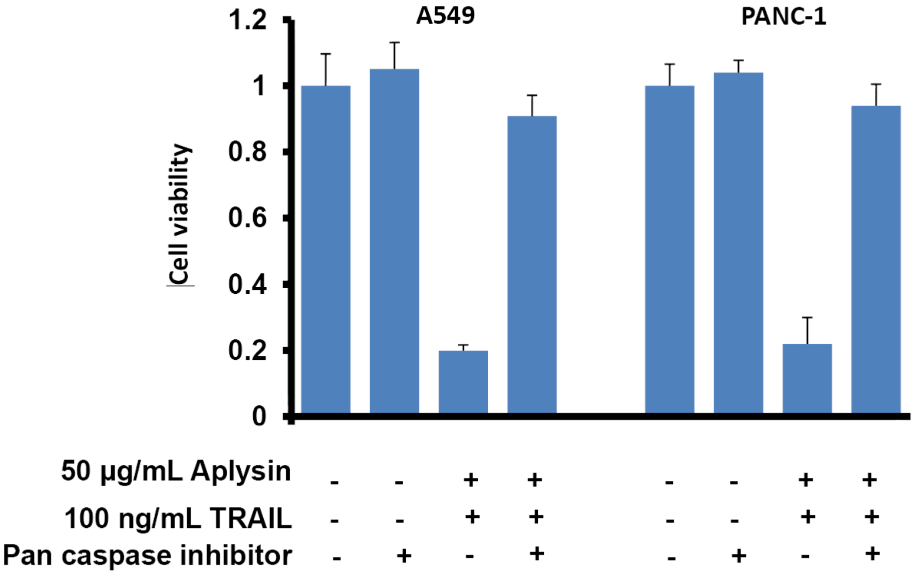

Figure S3. The expression of levels of survivin in Figure 5B was quantified using ImageJ software and shown as bars.

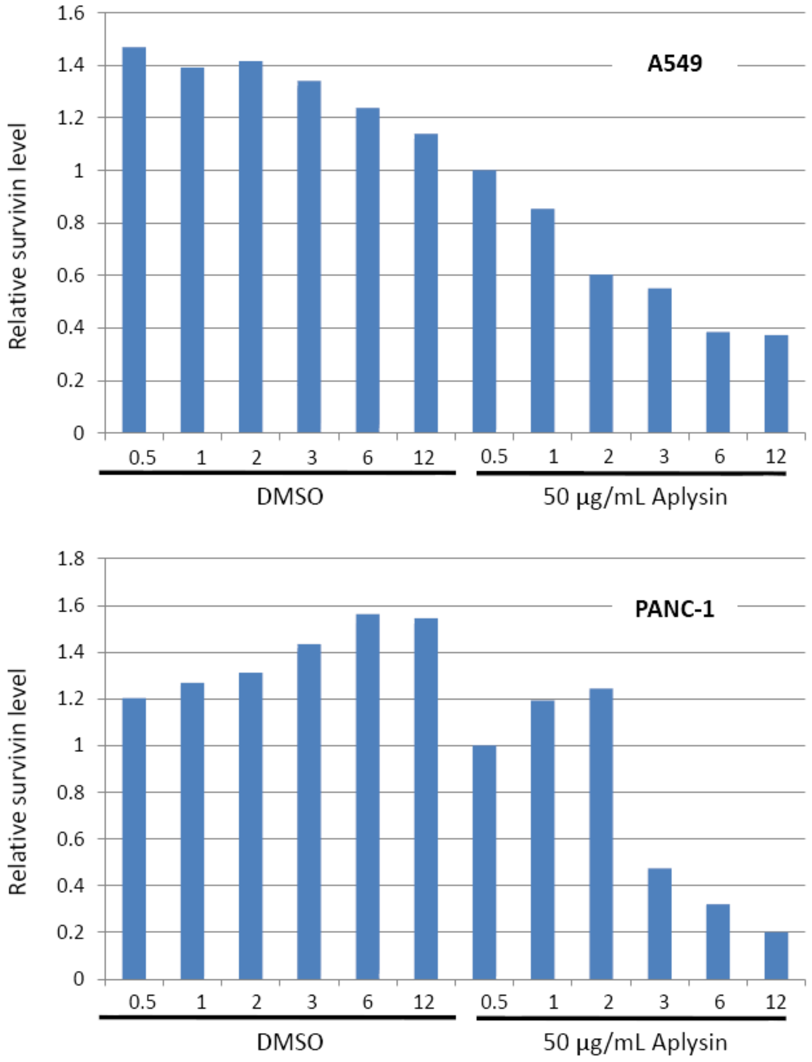

Figure S4. The expression of levels of p-p38 MAPK in Figure 7B was quantified using ImageJ software and shown as bars.

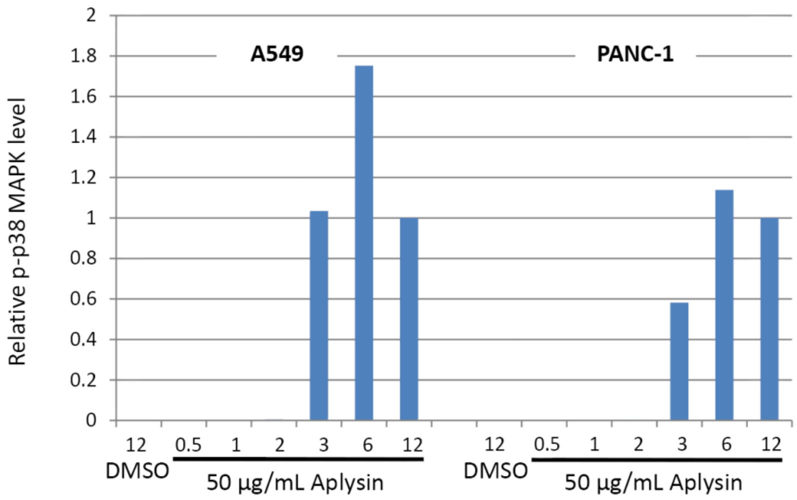

Figure S5. The expression of apoptosis inhibitors XIAP and cFLIP, cellular TRAIL receptor TRAIL-R1 and TRAIL-R2, and TRAIL decoy receoptor TRAIL-R3 and TRAIL-R4, were detected in A549 and PANC1 cells after 12 h treatment of Aplysin (50 μg/mL).

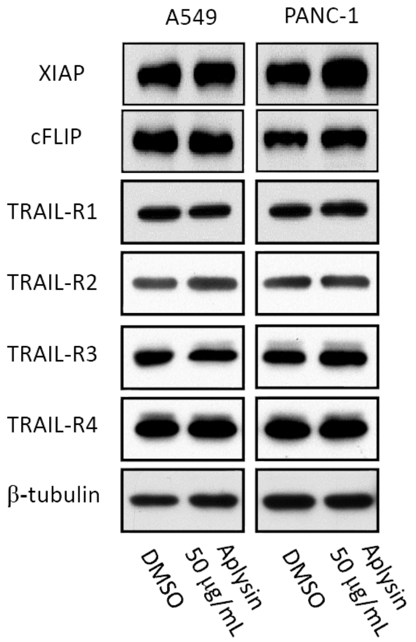

Figure S6. The expression levels of the pro-forms of caspase 3, 8 and 9 in Figures 4A (**A**) and 6C (**B**) were shown here.

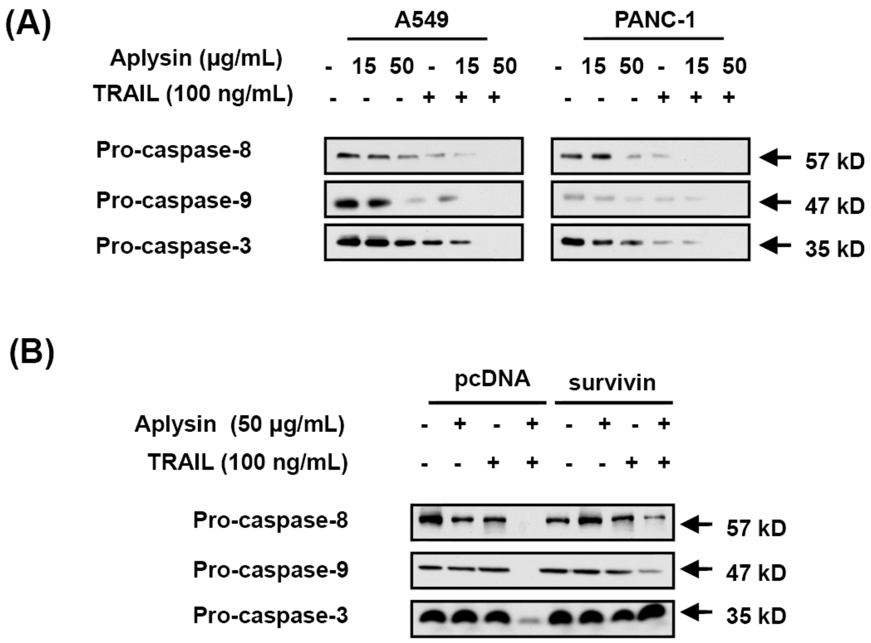

© 2014 by the authors; licensee MDPI, Basel, Switzerland. This article is an open access article distributed under the terms and conditions of the Creative Commons Attribution license (http://creativecommons.org/licenses/by/3.0/).
